# Retraction: *Rickettsia* Species in African *Anopheles* Mosquitoes

**DOI:** 10.1371/journal.pone.0348643

**Published:** 2026-05-06

**Authors:** The PLOS One Editors

The *PLOS One* Editors retract this article [1,2] due to concerns about compliance with the PLOS Human Subjects Research policy.

Following the publication of the article and Expression of Concern [1,2], PLOS investigated concerns pertaining to the reported ethical approval and the article’s adherence to *PLOS One’s* research ethics policies.

Specifically, the article reports the use of human landing catches conducted by French forces in rural or urban areas in Côte d’Ivoire (2004), Gabon (2007), and Senegal (2005–2007). The Ethics Statement reports that the study protocol was approved by the Marseille II Ethics Committee (Advice N. 02/81, 12/13/2002) and that an authorization of medical research was obtained also for a period of two years with the Ministry of Health and Public Hygiene in charge of Family and Women Promotion, Gabon (Decision No. 001085 of September 2008). The article does not report any ethics approval from Côte d’Ivoire or Senegal.

The authors did not respond to the journal’s request for additional information and ethics approval documentation.

A representative from the Aix-Marseille Université stated that the institute disagrees with the retraction decision and stated that no authorization is required to catch mosquitos in any country in the world, and that the ethics statement in the article appears erroneous.

PLOS does not consider that the institute’s response resolves the concerns pertaining the article’s compliance with the PLOS Human Subjects Research policy.

DR responded but expressed neither agreement nor disagreement with the editorial decision. CS, FP, MON, and PR either did not respond directly or could not be reached.

## References

[1] Socolovschi C, Pages F, Ndiath MO, Ratmanov P, Raoult D. RETRACTED: *Rickettsia* species in African *Anopheles* mosquitoes. PLoS One. 2012;7(10):e48254. doi: 10.1371/journal.pone.0048254 23118963 PMC3484133

[2] The *PLOS ONE* Editors. Expression of Concern: *Rickettsia* Species in African *Anopheles* Mosquitoes. PLoS One. 2022;17(12):e0278001. doi: 10.1371/journal.pone.0278001 36512594 PMC9746942

